# Comparison of Endoscopic Radiofrequency Ablation and Argon Plasma Coagulation in Patients with Gastric Low-Grade Intraepithelial Neoplasia: A Large-Scale Retrospective Study

**DOI:** 10.1155/2022/2349940

**Published:** 2022-06-22

**Authors:** Nanjun Wang, Ningli Chai, Longsong Li, Huikai Li, Yaqi Zhai, Xiuxue Feng, Shengzhen Liu, Wengang Zhang, Enqiang Linghu

**Affiliations:** Department of Gastroenterology, The First Medical Center of Chinese PLA General Hospital, Beijing, China

## Abstract

**Background:**

Gastric low-grade intraepithelial neoplasia (LGIN) is a precancerous lesion of gastric cancer. Endoscopic therapies represented by radiofrequency ablation (RFA) and argon plasma coagulation (APC) have been applied to treat gastric LGIN in recent years. However, no comparative study examining the effectiveness and safety profiles of RFA and APC has been reported.

**Methods:**

A single-center, large-scale, retrospective study, including 73 and 50 patients treated with RFA and APC, respectively, was conducted in the First Medical Center of Chinese PLA General Hospital from October 2015 to October 2020, with a two-year follow-up. Effectiveness, complications, operative factors, and other data were assessed.

**Results:**

At 2 years of follow-up, cure, relapse, recurrence, and progression rates were 90.4%, 9.6%, 9.6%, and 2.7% in the RFA group, respectively, versus 90%, 10%, 12%, and 4% in the APC group, respectively, with no statistically significant differences between the two groups (all *p* > 0.05). However, the mean lesion size was significantly larger in the RFA group (2.6 ± 1.0 cm) than in the APC group (1.5 ± 0.6 cm) (*p* < 0.001); there was also a significant difference in the composition ratio of large lesions between the two groups (*p* < 0.001). No serious postoperative complications showed in either group, and the abdominal pain was the most common symptom in the short term after surgery.

**Conclusions:**

RFA and APC are both safe and effective destructive therapies for gastric LGIN. RFA is more suitable for flat and large lesions, while APC is more suitable for small lesions, especially those with slight local uplift or depression. An intraoperative submucosal injection is expected to be an effective method for relieving postoperative abdominal pain.

## 1. Introduction

A few decades ago, the World Health Organization (WHO) introduced the notion of intraepithelial neoplasia in the recent classification of digestive system tumors, referring to the Vienna International Consensus [1–3]. In the latter classification, gastric mucosal intraepithelial neoplasia can be divided into high-grade intraepithelial neoplasia (HGIN) and low-grade intraepithelial neoplasia (LGIN), depending on the extent of cellular and structural atypia. It is well known that gastric mucosa intraepithelial neoplasia is a precancerous lesion of gastric cancer. If precancerous lesions can be eliminated, gastric cancer could be effectively prevented. With a deepened understanding of the disease progression and the improvement of therapeutic tools, a consensus has been formed on the clinical management of HGIN, namely; timely endoscopic treatment or surgery is the preferred option [4, 5]. Although no consensus has been reached on the principles of LGIN management, some guidelines [6, 7] have recommended aggressive endoscopic treatment for long-term gastric LGIN due to the potential progression to gastric cancer [8–11].

As LGIN is at an earlier stage than HGIN in precancerous lesions, endoscopic resection therapies are also feasible, including endoscopic mucosal resection (EMR) and endoscopic submucosal dissection (ESD), as confirmed by previous reports [12, 13]. However, endoscopic resection is a high-level treatment endoscopic technique, with a long learning period, relatively difficult operation, complex postoperative management, high cost, and potential serious complications in the perioperative period [14]. The above disadvantages have limited the further application of endoscopic resection therapy in LGIN.

Correspondingly, radiofrequency ablation (RFA) and argon plasma coagulation (APC), the two most commonly used methods in damage therapy, have been preliminarily reported in clinical studies for the treatment of gastric LGIN in recent years [15–19]. They have the advantages of simple operation, low cost, low risk, and outpatient treatment, gradually showing good clinical application prospects.

No study has reported the differences in effectiveness and complication among damage therapies for gastric LGIN. For this purpose, we designed this retrospective study to compare RFA and APC in the treatment of gastric LGIN.

## 2. Methods

### 2.1. Patients

The records of 123 consecutive patients administered RFA or APC for gastric LGIN in The First Medical Center of Chinese PLA General Hospital between October 2015 and October 2020 and followed up for more than 2 years were reviewed in this single-center retrospective study. Among them, 73 and 50 patients received RFA and APC, respectively. All the patients provided written informed consent for the procedure, and the study was reviewed and approved by the Ethics Committee of Chinese PLA General Hospital.

### 2.2. Inclusion and Exclusion Criteria

The inclusion criteria were as follows: (a) treatment with RFA (macroscopic type 0-II lesions according to the Paris classification [20]) or APC (no specific limitation); (b) 18–85 years of age; and (c) according to the WHO standards [1], confirmed LGIN by preoperative biopsy and HGIN and early gastric cancer (EGC) ruled out. The exclusion criteria were as follows: (a) HGIN or EGC confirmed or not excluded by biopsy before the operation; (b) a history of gastric surgery; (c) patients with severe cardiopulmonary disease who could not undergo anesthesia; (d) patients with advanced chronic liver disease or other serious systemic diseases who could not tolerate the operation; and (e) patients with coagulation dysfunction or unable to complete follow-ups as required.

### 2.3. Instruments and Procedures for RFA and APC

The BARRX System (Covidien GI Solutions, Sunnyvale, CA, United States) was used for RFA, and the argon plasma coagulation unit (APC 300, ERBE Elektromedizin, Tübingen, Germany) was used for APC. A disposable injector (NM-200L-0425, Olympus, Tokyo, Japan) with normal saline solution was used for submucosal injections. An accessory of the BARRX System (Covidien TTS-1100, 60RFA Conduit 909300, Sunnyvale, CA, United States) was used for lesion ablation. Hemostatic forceps (FD-410 LR, Olympus Medical, Tokyo, Japan) were used to prevent hemorrhage and perforation. Other equipment and accessories included a high-frequency generator (ICC-200, ERBE Elektromedizin, Tübingen, Germany), gastroscopes (GIF-Q260 J, GIF-H260Z, GIF-HQ290, Olympus Medical, Tokyo, Japan), and carbon dioxide gas with a CO_2_ insufflator (UCR, Olympus Medical, Tokyo, Japan).

The procedure applied for RFA has been reported previously [17]. After the lesions were found by routine gastroscopy, they were further examined by magnifying endoscopy (ME) combined with narrow-band imaging (NBI) to determine the size and range. Next, an RFA electrode was attached to the lesion surface with the assistance of endoscopy. The output power for RFA was set to 57 W, and the energy density was 15 J/cm^2^. After ablation, the lesion surface showed white coagulation and necrosis. Before the next ablation, the coagulated necrotic tissue on the surface was removed with RFA electrodes. RFA was repeated three times for each lesion to ensure complete ablation. In addition, submucosal injection could be administered to lesions, which was beneficial for the procedure, especially in case of difficult lesions. Other details of the RFA procedure were described in our previous study [17].

**Figure 1:**
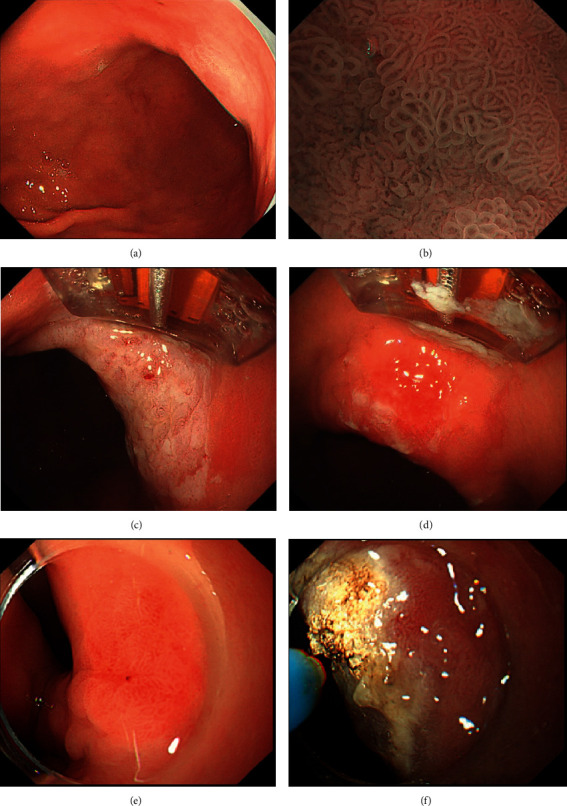
Radiofrequency ablation and argon plasma coagulation procedures for gastric low-grade intraepithelial neoplasia. (a) White-light imaging of the lesion. (b) Magnifying endoscopy with narrow-band imaging of the lesion (strong magnification). (c) After ablation, the surface of the lesion showed white coagulation and necrosis. (d) After scraping off the necrotic mucosal tissue on the surface. (e) White-light imaging of another lesion (reversed view). (f) After argon plasma coagulation, the surface of the lesion showed light yellow and brown-black coagulation and necrosis.

The procedure applied for APC was simpler than that of RFA. First, it was also necessary to re-evaluate the lesions with ME combined with NBI. Next, with the help of endoscopy, an argon plasma catheter was placed close to the lesion surface and cauterized in a subcontact state. Unlike RFA, we set the output power for APC to 35 W. After APC, the lesion surface showed white, light yellow, or brown-black coagulation areas and necrosis. Finally, the procedure was considered to be completed after confirming that the treatment had completely covered the lesion area. It is important to note that APC does not require retreatment of the same lesion area except for a local omission. Similar to RFA, submucosal injection was used in APC during the procedure. Other details of the APC procedure were described in our previous study [21]. The procedures of RFA and APC, performed by three experienced gastrointestinal endoscopists (E. Q. Linghu, N. L. Chai, and N. J. Wang), are shown in Figure 1.

### 2.4. Additional Treatments and Follow-Up

Postoperative management and follow-up were performed according to the previous study [17].

Each patient fasted for 4–6 h after the procedure. Then, a liquid or semiliquid diet was provided, followed by gradual transition to a normal diet. At the same time, patients were administered a proton pump inhibitor (PPI) and a mucosal protectant for 1 month postsurgically. Moreover, we explained the Wong–Baker FACES Pain Rating Scale [22] to the patients who were each provided a form for self-recording the daily pain score in the first month after RFA or APC. The forms were returned 3 months after the patient returned to our hospital for the first review.

All patients were required to return to our hospital for follow-up at 3 months, 6 months, 1 year, 2 years, 3 years, 4 years, and 5 years after surgery. Patients were examined by gastroscopy, and biopsies were performed in the original treatment area and other suspected areas. Pathological findings were used to determine whether the treatment was effective, as well as to assess relapse, recurrence, and progression. In this research, according to the study design, the data of patients followed up for 2 years after the operation were used as the evaluation criteria. The following definitions were used. (1) LGIN disappearance in the original treatment area indicated by pathological biopsy indicated a curative effect. (2) LGIN presence in the original treatment area indicated relapse. (3) LGIN presence in a nontreatment area was indicated recurrence. (4) HGIN or EGC presence in the original treatment area indicated disease progression.

On the other hand, perioperative complications and adverse events, including bleeding, perforation, infection, and postoperative abdominal pain, were used to assess the safety of each operation.

### 2.5. Statistical Analysis

Data analysis was performed using SPSS Statistics for Windows, version 26.0 (IBM, Armonk, NY, USA). The data were retrospectively collected, and the procedural parameters were compared. Measurement data were expressed as the mean ± standard deviation or the median with range, whereas numerical data were described as frequency and percentage and were compared by the *χ*2 test or Fisher's exact test. The Chi-square test was performed to compare categorical variables. The measurement data were analyzed by the t-test and one-way analysis of variance or the rank-sum test according to normality. *p* < 0.05*p* < 0.05 was considered statistically significant.

## 3. Results

### 3.1. Clinical Characteristics and Procedure-Related Parameters

A total of 123 patients with the mean age of 56.9 (range: 22–80) years were enrolled in the study (77 males and 46 females). Seventy-three patients received RFA and 50 underwent APC. Of all patients, 59 had a course of disease longer than 1 year, including 33 and 26 in the RFA and APC groups, respectively. In the RFA group, there were 1, 4, 29, and 39 lesions located in the gastric fundus, body, angle, and antrum, respectively, versus 1, 3, 17, and 29 cases in the APC group, respectively. The average operation time of the two procedures was about 15.2 minutes and 14.7 minutes, respectively. During the operation, 32 patients in the RFA group and 20 in the APC group received submucosal injection. In addition, there were 15 *Helicobacter pylori* (*H. pylori*) infection and 27 atrophic gastritis cases in the RFA group compared with that of 8 and 14 cases in the APC group, respectively. There were no statistically significant differences between the two groups in gender, age, disease course, lesion location, operation time, proportion of submucosal injection, *H. pylori* infection, and atrophic gastritis (all *p* > 0.05). However, the mean lesion size was significantly larger in the RFA group (2.6 ± 1.0 cm) than in the APC group (1.5 ± 0.6 cm) (*p* < 0.001). Moreover, there were also significant differences in the composition ratio of large lesions between the two groups (*p* < 0.001). All clinical characteristics and procedure-related parameters of both groups are shown in Table 1.

### 3.2. Therapeutic Effectiveness and Long-Term Outcomes

All patients in both groups completed 2 years of postoperative follow-up, including endoscopic and symptomatic examinations. At 2 years of follow-up, the cure, relapse, recurrence, and progression rates were 90.4%, 9.6%, 9.6%, and 2.7% in the RFA group, respectively, versus 90%, 10%, 12%, and 4% in the APC group, respectively. However, these differences were not statistically significant between the two groups (all *p* > 0.05). Some patients with relapse and recurrence were treated with RFA or APC again, while others were followed up for observation. The follow-up of these patients is still ongoing, and no case with further progression has been recorded yet. Additional ESD therapy was performed in all 4 patients with HGIN disease progression. The short-term follow-up results of all 4 patients indicated curative resection, and the long-term follow-up is still in progress. The specific data are also shown in Table 1.

### 3.3. Procedure-Related Adverse Events

As shown in Table 1, postoperative abdominal pain occurred in both groups, and the difference was not statistically significant (42, 57.5% vs. 31, 62.0%, *p*=0.620). Most of the pain occurred within 14 days postoperatively. All these patients experienced gradual relief of symptoms after taking PPIs and mucosal protectants. Meanwhile, in the RFA group, abdominal pain was developed in 10 of the 32 patients administered submucosal injection, compared to 32 of the 41 who did not receive submucosal injection (*p* < 0.001). Similarly, in the APC group, 7 in 20 patients administered submucosal injection developed abdominal pain, while 6 in 30 individuals not administered submucosal injection had no obvious abdominal pain, showing a significant difference between the two groups (*p*=0.001). Overall, only 17 of the 52 patients administered submucosal injection developed abdominal pain, while up to 56 of the 71 not administered submucosal injection developed abdominal pain, with a statistically significant difference (*p* < 0.001). These data are presented in detail in Table 2. In addition, no perioperative bleeding, perforation, infection, or other serious complications occurred in any of the 123 patients.

## 4. Discussion

Recently, endoscopic RFA and APC have been applied for the clinical treatment of gastric LGIN, with their working principles described in several previous reports [15–19, 21]. RFA causes the movement of charged particles in tissues to generate heat through the action of high-frequency alternating current to evaporate water inside and outside the cells, which dry, shrink, and fall off, resulting in aseptic necrosis. Furthermore, the output power and energy density of each RFA are controlled and do not increase with the operation time. Unlike RFA, APC is a noncontact damage treatment, which exerts effects by spraying ionized argon gas onto the target mucosal surface, thereby transferring high-frequency electrical energy to tissues and utilizing thermal effects to deactivate and dry the tissue and to cause coagulation and necrosis. In general, both procedures achieve the goal of treating lesions by inducing local damage.

As previously mentioned, a few studies have preliminarily explored the clinical treatment effectiveness of RFA and APC in gastric LGIN. However, no reports have compared large clinical samples between RFA and APC, and this study filled this gap.

First, we compared clinical characteristics and procedure-related parameters between the two groups. In this study, no statistically significant differences were found between the RFA and APC groups in terms of the gender, age, disease course, lesion location, operation time, proportion of submucosal injection, and mucosal background (*H. pylori* infection and atrophic gastritis). However, the overall lesions were larger in the RFA group than in the APC group, which may be related to divergent working principles of RFA and APC. For the former method, using an electrode patch for ablation can treat larger lesion areas, while the latter is more favorable for treating smaller lesions because of the point-like cauterization. Similarly, flat lesions were selected for RFA because the electrode patch used for ablation is flat, while the treatment method in APC is point-like cauterization so that APC is also suitable for swelling or sunken lesions. This caused a significant difference in the composition ratio of large lesions between the two groups. However, it should be noted that HGIN or EGC more likely occurs in nonflat lesions, especially in depressed ones. Therefore, special attention should be paid to preoperative evaluation and screening.

Secondly, both RFA and APC showed good clinical effectiveness for gastric LGIN, with effectiveness rates in both groups surpassing 90% in the 2-year follow-up period, which preliminarily suggests the damage therapy of gastric LGIN is simple and efficient. However, there were still some patients with postoperative relapse and recurrence. The results showed *H. pylori* infection and/or atrophic gastritis were present in 21 of all the 25 relapse or recurrence cases. Meanwhile, patients with a disease course longer than 1 year also accounted for a high proportion of relapse or recurrence cases (76%, 19/25). According to the results, *H. pylori* infection and atrophic gastritis might change the overall state and microenvironment of the gastric mucosa to some extent, and a longer disease course might further exacerbate these changes, which are all possible causes of LGIN relapse or recurrence. This finding corroborated previous studies [23, 24] because *H. pylori* predisposes the mucosa to intestinal metaplasia and the odds of intraepithelial neoplasia are higher in atrophic and intestinal metaplasia than in the normal mucosa. Meanwhile, we noted that more than half of lesions were concentrated in the gastric antrum, which might be associated with the early occurrence of mucosal atrophy in the gastric antrum and its susceptibility to *H. pylori*. This also supported our conclusions from another aspect.

In addition, during the follow-up period, 4 patients in both groups had disease progression, from LGIN to HGIN. A review of previous studies showed that lesion size >1 cm, erythema, erosion, ulceration, nodular changes on the lesion surface, and significant depression of the lesion are all risk factors for progression from LGIN to HGIN or EGC [25, 26]. Lesion sizes in our 4 cases were all over 1 cm; of these, 3 cases were accompanied by surface erythema and the remaining had mild erosion. At the same time, none of the 4 cases had ulceration or obvious depression. We reviewed the images obtained by preoperative magnification endoscopy again and found no significant loss of surface microstructures or microvessels, and these lesions were still included in patient screening. This also indicated that the existing endoscopic screening theory of EGC might still need to be further improved. We look forward to carrying out further research in this field in the future. On the other hand, although we carried out sufficient endoscopic assessment and localization, preoperative biopsy still could not fully reflect the overall situation of the lesion, and there is potential bias. This might allow some lesions already of the HGIN or EGC type to be included in the study. This is another possible cause of postoperative pathological escalation.

Thirdly, in terms of postoperative adverse events, more than half of patients in both groups experienced short-term abdominal pain after surgery. Further analysis indicated patients administered submucosal injection during surgery had a lower incidence of postoperative abdominal pain compared with those not administered submucosal injection in the RFA, APC, or whole cohort, corroborating a previous study [27]. The mechanism might involve the protection and thermal partition of the deep muscle tissue by using the liquid pad formed by submucosal injection. Therefore, the submucosal injection was recommended during the damage therapy. This needs to be clarified in subsequent, larger randomized controlled studies. Furthermore, no serious complications such as bleeding, perforation, and infection occurred in either group during the perioperative period, and the patients had satisfactory safety. However, the risk of perforation in APC was reported previously [28]. The main cause of perforation was deep burning during the operation. Therefore, it is very important to maintain the subcontact state between the argon plasma catheter and the lesion surface. For medical centers preparing to carry out this treatment, it is advisable for endoscopists with relatively long treatment experience to complete the procedure.

Last but not the least, operation times in both groups were very short, about 15 minutes. In general, RFA and APC can be performed by endoscopists as long as they are skilled in gastroscopy, which results in significantly reduced cost and the learning curve for surgical training in RFA and APC. Meanwhile, both procedures can be performed on an outpatient basis, which effectively saves medical resources. All these advantages constitute the basis for the clinical application and promotion of these techniques in the future.

This study had some limitations. Firstly, this was a single-center retrospective study with a certain inherent bias. In addition, improving the accuracy of preoperative evaluation remains a clinical difficulty that needs further investigation. Furthermore, it is also urgent to carry out large-sample randomized controlled clinical studies on submucosal injection for the relief of postoperative abdominal pain.

## 5. Conclusion

RFA and APC are both safe and effective damage therapies for gastric LGIN. RFA is more suitable for flat and large lesions, while APC is more suitable for small lesions, especially those with slight local uplift or depression. Intraoperative submucosal injection is expected to be an effective tool for relieving postoperative abdominal pain. As simple and efficient endoscopic treatment techniques for gastric LGIN, both tools are worthy of further clinical promotion.

## Figures and Tables

**Table 1:** Baseline characteristics, procedure-related parameters, and follow-ups.

	RFA group (*n* = 73)	APC group (*n* = 50)	*p* value
Sex, male/female (*n*)	45/28	32/18	0.791
Age, mean ± standard deviation (years)	57.1 ± 10.8	56.5 ± 11.2	0.759
A course of disease, *n* (%)			0.459
<1 year	40 (54.8)	24 (48.0)	
>1 year	33 (45.2)	26 (52.0)	
Macroscopic type, *n* (%)			<0.001
0-I	0 (0)	25 (50.0)	
0-II	73 (100)	23 (46.0)	
0-III	0 (0)	2 (4.0)	
Ulceration	0	0	
Location of lesions, *n* (%)			0.917
Gastric fundus	1 (1.4)	1 (2.0)	
Gastric body	4 (5.5)	3 (6.0)	
Angle of the stomach	29 (39.7)	17 (34.0)	
Gastric antrum	39 (53.4)	29 (58.0)	
Size of lesions, mean ± standard deviation (cm)	2.6 ± 1.0	1.5 ± 0.6	<0.001
Operating time, mean ± standard deviation (min)	15.2 ± 1.8	14.7 ± 1.8	0.133
Submucosal injection, *n* (%)	32 (43.8)	20 (40.0)	0.672
Helicobacter pylori infection, *n* (%)			0.525
Yes	15 (20.5)	8 (16.0)	
No	58 (79.5)	42 (84.0)	
Atrophy, *n* (%)			0.299
Yes	27 (37.0)	14 (28.0)	
No	46 (63.0)	36 (72.0)	
2-year follow-up, *n* (%)			
Curative	66 (90.4)	45 (90.0)	1.000
Relapse	7 (9.6)	5 (10.0)	1.000
Recurrence	7 (9.6)	6 (12.0)	0.669
Progression	2 (2.7)	2 (4.0)	1.000
Abdominal pain, *n* (%)	42 (57.5)	31 (62.0)	0.620

**Table 2:** Submucosal injection for postoperative abdominal pain relief.

	Submucosal injection group	Non-submucosal injection group	*p* value
Abdominal pain relief ratio in the RFA group	22/32 (68.8)	9/41 (22.0)	<0.001
Abdominal pain relief ratio in the APC group	13/20 (65.0)	6/30 (20.0)	0.001
Total	35/52 (67.3)	15/71 (21.1)	<0.001

## Data Availability

All data obtained or analyzed during this work are included within the article.

## Abbreviations

WHO: World Health Organization
HGIN: High-grade intraepithelial neoplasia
LGIN: Low-grade intraepithelial neoplasia
RFA: Radio frequency ablation
APC: Argon plasma coagulation
EGC: Early gastric cancer
ME: Magnifying endoscopy
NBI: Narrow-band imaging
PPI: Proton pump inhibitor
H. pylori: *Helicobacter pylori*.

## References

[1] Watterson J. D., Mahoney J. E., Futter N. G., Gaffield J. (1998). Iatrogenic ureteric injuries: approaches to etiology and management. *Canadian journal of surgery. Journal canadien de chirurgie*.

[2] Dixon M. F. (2002). Gastrointestinal epithelial neoplasia: Vienna revisited. *Gut*.

[3] Schlemper R. J., Riddell R. H., Kato Y. (2000). The Vienna classification of gastrointestinal epithelial neoplasia. *Gut*.

[4] Ono H., Yao K., Fujishiro M. (2021). Guidelines for endoscopic submucosal dissection and endoscopic mucosal resection for early gastric cancer (second edition). *Digestive Endoscopy*.

[5] Expert group of Beijing Municipal Science and Technology Commission Major Project (2019). Expert consensus on endoscopic standardized resection of early gastric cancer (2018, Beijing). *Chinese Journal of Digestive Endoscopy*.

[6] ASGE Standards of Practice Committee, Evans J. A., Chandrasekhara V. (2015). The role of endoscopy in the management of premalignant and malignant conditions of the stomach. *Gastrointestinal Endoscopy*.

[7] Division of Digestive Endoscopy of Beijing Medical Association (2019). Expert consensus on standardized diagnosis and treatment of gastric low-grade intraepithelial neoplasia (2019, Beijing). *Chinese Journal of Gastrointestinal Endoscopy (Electronic Edition)*.

[8] Kim M. K., Jang J. Y., Kim J.-W. (2014). Is lesion size an independent indication for endoscopic resection of biopsy-proven low-grade gastric dysplasia?. *Digestive Diseases and Sciences*.

[9] Srivastava A., Lauwers G. Y. (2008). Gastric epithelial dysplasia: the Western perspective. *Digestive and Liver Disease*.

[10] Raftopoulos S. C., Kumarasinghe P., De Boer B. (2012). Gastric intraepithelial neoplasia in a Western population. *European Journal of Gastroenterology and Hepatology*.

[11] Gregorio C. D., Morandi P., Fante R., De Gaetani C. (1993). Gastric dysplasia. A follow-up study. *American Journal of Gastroenterology*.

[12] Park Y.-M., Cho E., Kang H.-Y., Kim J.-M. (2011). The effectiveness and safety of endoscopic submucosal dissection compared with endoscopic mucosal resection for early gastric cancer: a systematic review and metaanalysis. *Surgical Endoscopy*.

[13] Cao Y., Liao C., Tan A., Gao Y, Mo Z, Gao F (2009). Meta-analysis of endoscopic submucosal dissection versus endoscopic mucosal resection for tumors of the gastrointestinal tract. *Endoscopy*.

[14] Kim S. Y., Sung J. K., Moon H. S. (2012). Is endoscopic mucosal resection a sufficient treatment for low-grade gastric epithelial dysplasia?. *Gut and Liver*.

[15] Baldaque-Silva F., Cardoso H., Lopes J., Carneiro F., Macedo G. (2013). Radiofrequency ablation for the treatment of gastric dysplasia. *European Journal of Gastroenterology and Hepatology*.

[16] Leung W. K., Tong D. K., Leung S. Y. (2015). Treatment of gastric metaplasia or dysplasia by endoscopic radiofrequency ablation: a pilot study. *Hepato-Gastroenterology*.

[17] Wang N.-J., Chai N.-L., Tang X.-W., Li L.-S., Zhang W.-G., Linghu E.-Q. (2022). Clinical efficacy and prognostic risk factors of endoscopic radiofrequency ablation for gastric low-grade intraepithelial neoplasia. *World Journal of Gastrointestinal Oncology*.

[18] Lee K. M., Kim Y. B., Sin S. J. (2009). Argon plasma coagulation with submucosal saline injection for gastric adenoma on outpatient basis. *Digestive Diseases and Sciences*.

[19] Kim K. Y., Jeon S. W., Yang H. M. (2015). Clinical outcomes of argon plasma coagulation therapy for early gastric neoplasms. *Clinical Endoscopy*.

[20] (2003). The Paris endoscopic classification of superficial neoplastic lesions: esophagus, stomach, and colon: november 30 to December 1, 2002. *Gastrointestinal Endoscopy*.

[21] Meng M., Linghu E. Q., Wan D. (2014). Argon plasma coagulation for treatment of gastric low-grade intraepithelial neoplasia. *Chinese Journal of Gastrointestinal Endoscopy (Electronic Edition)*.

[22] Lawson S. L., Hogg M. M., Moore C. G. (2021). Pediatric pain assessment in the emergency department. *Pediatric Emergency Care*.

[23] Sung J. K. (2016). Diagnosis and management of gastric dysplasia. *The Korean Journal of Internal Medicine*.

[24] Liu Y. L., Cai Y. L., Chen S. (2020). Analysis of risk factors of gastric low-grade intraepithelial neoplasia in asymptomatic subjects undergoing physical examination. *Gastroenterol Res Pract*.

[25] Kim J.-W., Jang J. Y. (2015). Optimal management of biopsy-proven low-grade gastric dysplasia. *World Journal of Gastrointestinal Endoscopy*.

[26] Kang D. H., Choi C. W., Kim H. W. (2018). Predictors of upstage diagnosis after endoscopic resection of gastric low-grade dysplasia. *Surgical Endoscopy*.

[27] Niu X. T. (2016). *The Protective Effects of Submucosal Injection for Gastric Low-Grade Intraepithelial Neoplasia Treatment*.

[28] Watson J. P., Bennett M. K., Griffin S. M., Matthewson K. (2000). The tissue effect of argon plasma coagulation on esophageal and gastric mucosa. *Gastrointestinal Endoscopy*.

